# Supplementary material for: Effects of a Feed Sanitizer in Sow Diets on Sow and Piglet Performance
Source: Animals (Basel). 2025 Dec 16;15(24):3618. doi: 10.3390/ani15243618 (PMC12729499; doi:10.3390/ani15243618)
Supplement: Supplementary file 1 [file animals-15-03618-s001.zip › animals-4010684-supplementary.pdf]

animals

MDPI

Supplementary Materials

Table S1. *Escherichia coli* analysis of scour samples

Aerobic Culture	Samples Testing Positive for Culture Growth		Samples Testing Negative for Culture Growth	
	Control	Treatment[1]	Control	Treatment
Adhesion involved in diffuse adherence (AIDA-1)	0	0	13[2]	12[3]
Attaching and effacing factor (eae)	0	0	13	12
F18	0	0	13	12
F4 (K88)	0	0	13	12
F5 (K99)	0	0	13	12
F6 (987P)	0	0	13	12
Heat-labile enterotoxin (LT)	0	0	13	12
Heat-stable enterotoxin 1 (EAST - 1)	0	0	13	12
Heat-stable enterotoxin A (STa)	0	0	13	12
Heat-stable enterotoxin B (STb)	0	0	13	12
Porcine attaching and effacing-associated factor	0	0	13	12
Shiga toxin 2e (Stx2e)	0	0	13	12

[1]The feed sanitizer in this study was Termin-8, a formaldehyde and propionic based feed sanitizer. [2]During sampling, at least 4 scour samples were collected per farrowing crate from sows fed Control diets, and their piglets, and submitted for analysis to the Veterinary Diagnostic Laboratory (VDL). [3]During sampling, at least 2 scour samples were collected per farrowing crate from sows fed feed sanitizer diets, and their piglets, and submitted for analysis to the VDL..

 https://doi.org/10.3390/ani15243618

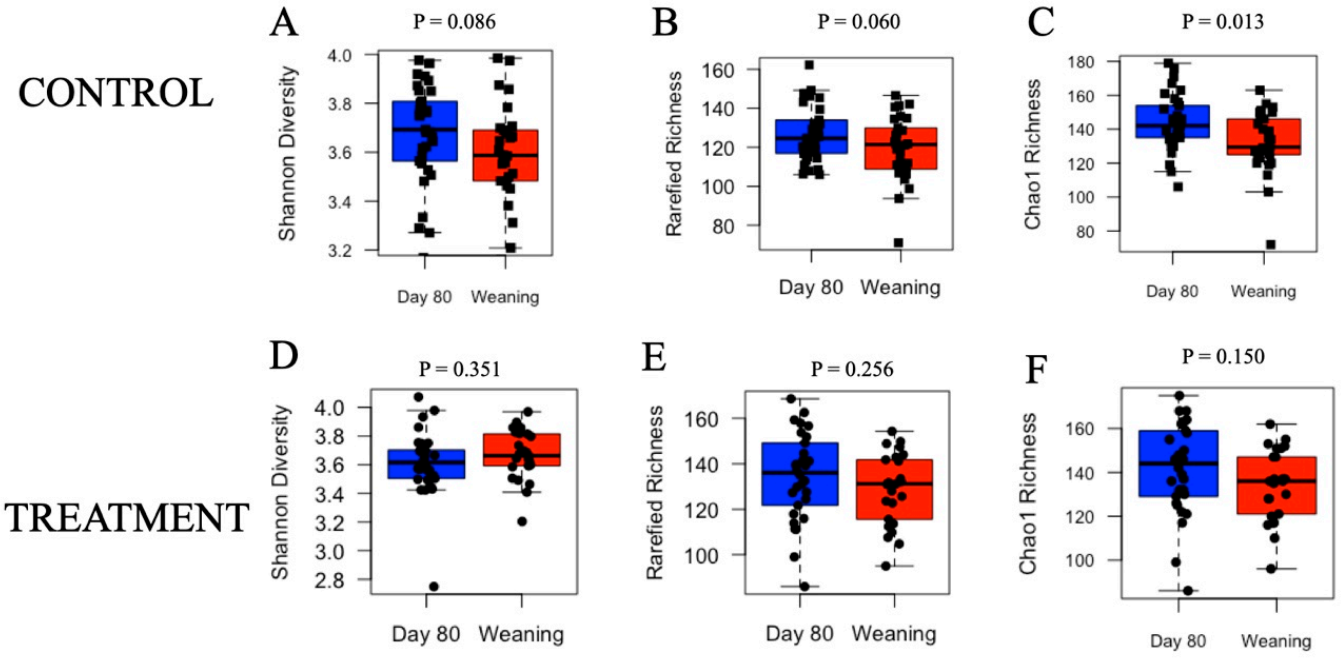

Figure S1. Bacterial alpha diversity between samples from sows fed Control diets (**a, b, c**; n=63) or Treatment diets **(d, e, f**; n=55**).** Samples were collected on day 80 of gestation before dietary treatments were imposed, shown in blue, and at weaning, shown in red. Significance was calculated with a t-test **(a, b, c, e, f)** and a Wilcoxon test (**d**).

Table S2. PERMANOVA analysis of microbial community composition from sows fed Control or Treatment diets

	Control			Treatment[1]		
**Factor**	*f*	*r*²	*p* **value**	*f*	*r*²	*p* **value**
**Group**[2]	3.517	0.040	**0.011**[3]	1.293	0.020	0.197
**Collection**[4]	23.854	0.271	**0.001**	13.371	0.202	**0.001**
**Pgroup**[5]	0.783	0.017	0.645	0.966	0.029	0.437
**Group × Collection**	1.934	0.022	0.067	2.088	0.032	0.057
**Group × Pgroup**	1.258	0.029	0.203	0.895	0.027	0.532
**Collection × Pgroup**	0.837	0.019	0.586	0.649	0.020	0.809
**Group × Collection × Pgroup**	0.920	0.021	0.496	0.684	0.650	0.769

[1]The feed sanitizer in this study was Termin-8, (Anitox Corp., Lawrenceville, GA, USA), a formaldehyde and propionic based feed sanitizer. [2]Farrowing group. [3]Variables with $p < 0.05$ were considered significant and are indicated in bold. [4]Time samples were collected in the study (gestation or weaning). [5]Parity group: parity 0-1, parity 2-3, and parity 4+

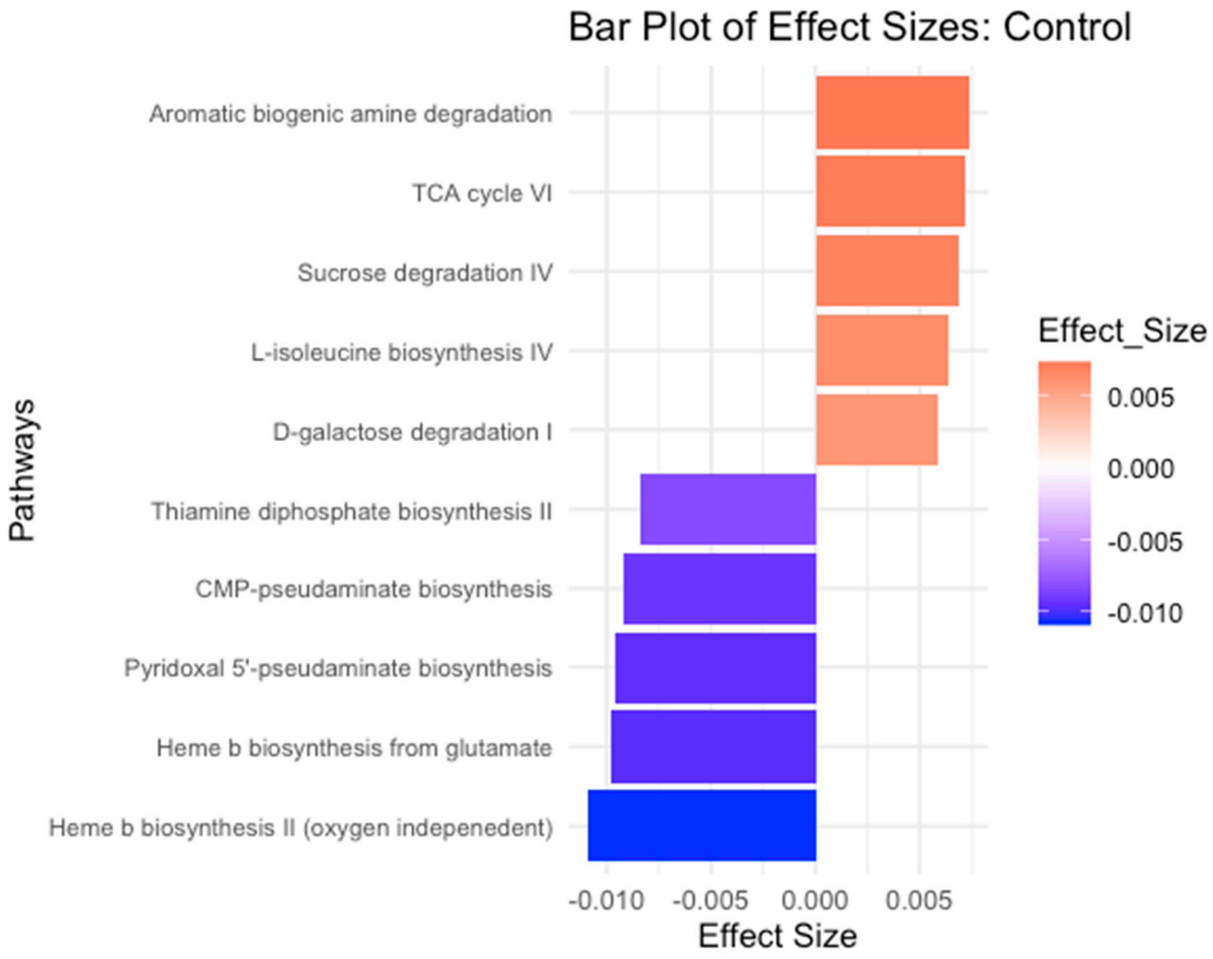

Figure S2 Effect sizes of significant microbial pathways in samples from sows fed Control (n=63) diets. Effect sizes of significant microbial pathways, as predicted by PICRUSt and determined by MaAsLin analysis ($p \leq 0.05$, $q \leq 0.005$). The top five pathways from each collection point are shown (red = pathways significant during lactation, blue = pathways significant during gestation).

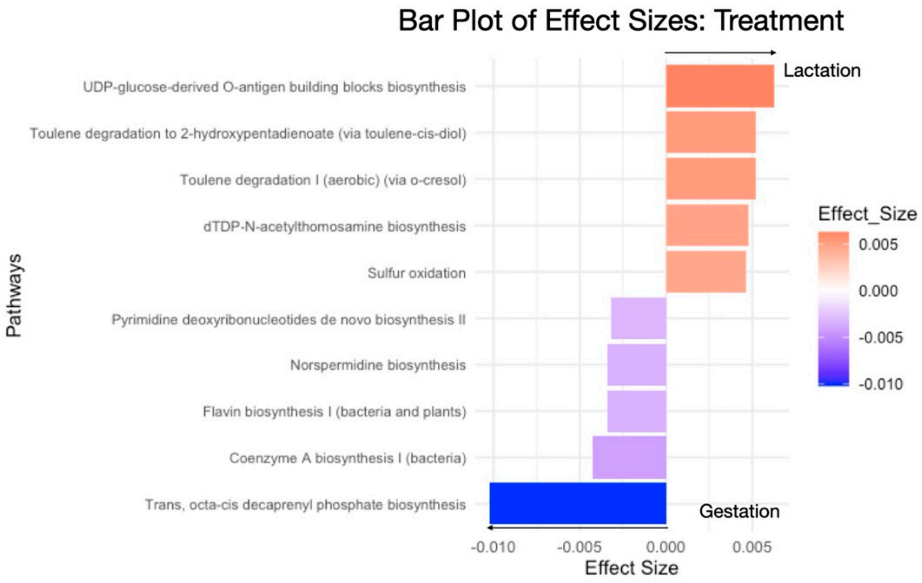

Figure S3. Effect sizes of significant microbial pathways in samples from sows fed Treatment (n=55) diets. Effect sizes of significant microbial pathways, as predicted by PICRUSt and determined by MaAsLin analysis ($p \leq 0.05$, $q \leq 0.005$). The top five pathways from each collection point are shown (red = pathways significant during lactation, blue = pathways significant during gestation).

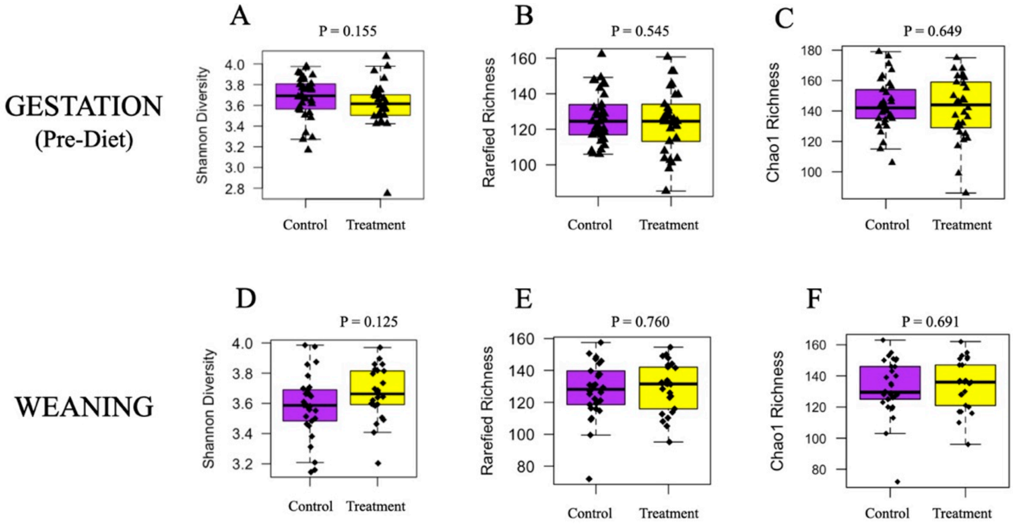

Figure S4. Bacterial alpha diversity between samples from sows during gestation **(a-c;** n=63**)** and sows sampled during weaning **(d-f;** n=55**).** Samples were then compared across treatments. Significance was calculated with a t-test **(c, d, e)** and a Wilcoxon test **(a, b f).**

Table S3. PERMANOVA analysis of vectors 2:3 of microbial community composition between sows at weaning fed the Control diet and sows fed the Treatment[1] diet

	Weaning		
**Factor**	***f***	***r*²**	***p* value**
**Treatment**	1944.2	0.752	**0.008**[2]
**Pgroup**[3]	-1877.7	-1.453	0.997
**Group**[4]	131.3	0.051	0.233
**Treatment × Pgroup**	2139.0	1.655	**0.002**
**Treatment × Group**	-730.6	-0.283	0.975
**Pgroup × group**	-671.0	-0.519	0.996
**Treatment × Pgroup × group**	1008.11	0.780	**0.006**

[1]The feed sanitizer in this study was Termin-8, (Anitox Corp., Lawrenceville, GA, USA) a formaldehyde and propionic based feed sanitizer. [2]Variables with $p < 0.05$ were considered significant and are indicated in bold. [3]Pgroup: parity 0-1, parity 2-3, and parity 4+. [4]Farrowing group.

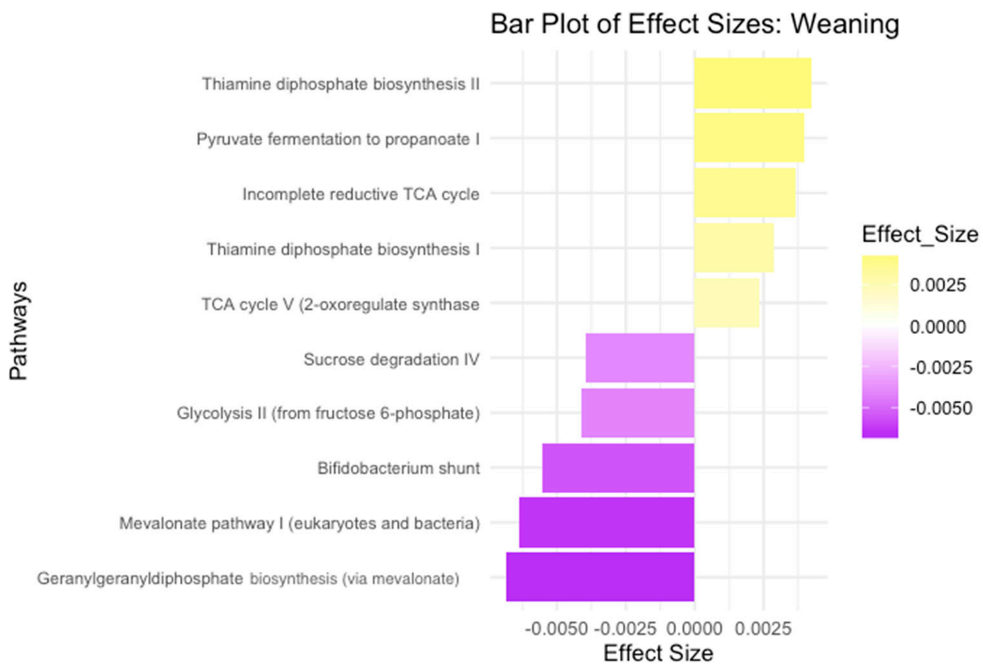

Figure S5. Effect sizes of significant microbial pathways at weaning in samples from sows fed Control (n=63) or Treatment (n=55) diets. Effect sizes of significant microbial pathways, as predicted by PICRUSt and determined by MaAsLin analysis ($p \leq 0.05$, $q \leq 0.005$). The top five pathways from each collection point are shown (yellow = pathways significant in Treatment sows, purple = pathways significant in Control sows).

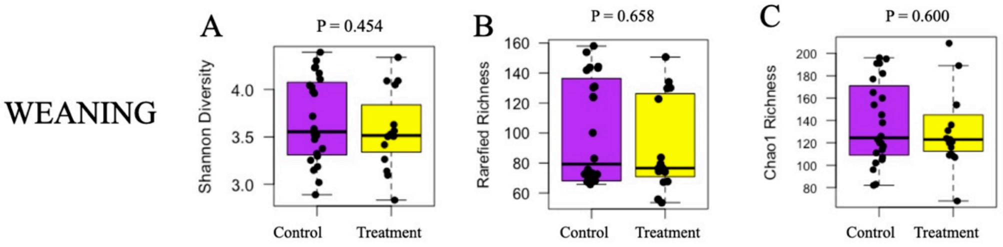

Figure S6. Bacterial alpha diversity metrics of piglets born to sows from Control diets (n=22; purple) or Treatment diets (n=17; yellow). Analysis included **(a)** Shannon diversity; **(b)** rarefied richness and **(c)** Chao1 richness. Significance was calculated with a t-test **(a, c)** and a Wilcoxon test **(b).**

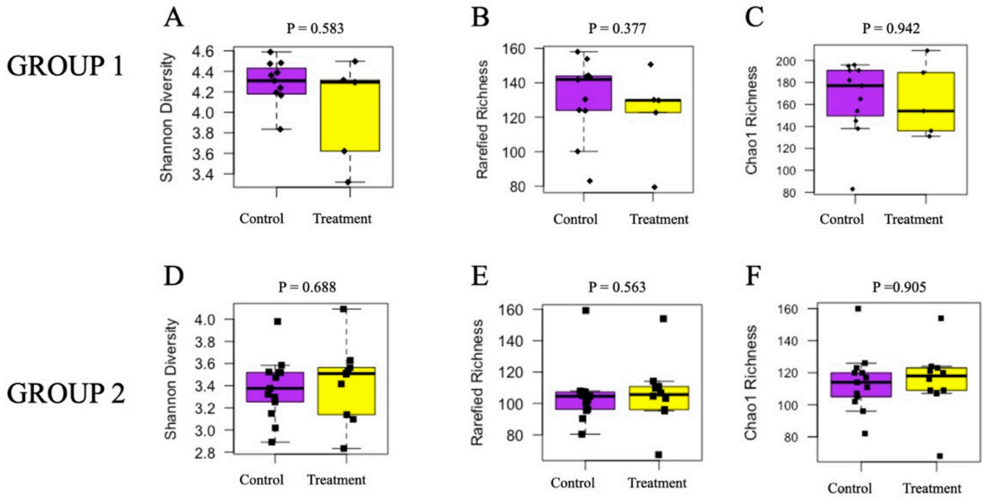

Figure S7. Bacterial alpha diversity between piglets sampled in Group 1 **(a, b**; n=17**)** and piglets sampled in Group 2 **(c, d;** n=22**)**. Samples were then compared across diets, (Control = purple, Treatment = yellow. Significance was calculated with a t-test **(c, d)** and a Wilcoxon test **(a, b, e, f).**
